# Optimization of process parameters for fabrication of electrospun nanofibers containing neomycin sulfate and *Malva sylvestris* extract for a better diabetic wound healing

**DOI:** 10.1080/10717544.2022.2144963

**Published:** 2022-11-21

**Authors:** Mohammed Monirul Islam, Varshini HR, Penmetsa Durga Bhavani, Prakash S. Goudanavar, N. Raghavendra Naveen, B. Ramesh, Santosh Fattepur, Predeepkumar Narayanappa Shiroorkar, Mohammed Habeebuddin, Girish Meravanige, Mallikarjun Telsang, Nagaraja Sreeharsha

**Affiliations:** a Department of Biomedical Sciences, College of Clinical Pharmacy, King Faisal University, Al-Ahsa, Saudi Arabia; b Sri Adichunchanagiri College of Pharmacy, Adichunchanagiri University, Karnataka, India; c Department of Pharmaceutics, Vishnu Institute of Pharmaceutical Education and Research, Telangana, India; d School of Pharmacy, Management and Science University, Selangor, Malaysia; e Department of Biomedical Sciences, College of Medicine, King Faisal University, Al-Ahsa, Saudi Arabia; f Department of Medicine, College of Medicine, King Faisal University, Al-Ahsa, Saudi Arabia; g Department of Pharmaceutical Sciences, College of Clinical Pharmacy, King Faisal University, Al-Hofuf, Al-Ahsa, Saudi Arabia; h Department of Pharmaceutics, Vidya Siri College of Pharmacy, Bangalore, India

**Keywords:** Neomycin sulfate, nanofibers, diabetics, wound healing, *Malva sylvestris*, optimization

## Abstract

Diabetes mellitus is one of the most concerning conditions, and its chronic consequences are almost always accompanied by infection, oxidative stress, and inflammation. Reducing excessive reactive oxygen species and the wound’s inflammatory response is a necessary treatment during the acute inflammatory phase of diabetic wound healing. *Malva sylvestris* extract (MS) containing nanofibers containing neomycin sulfate (NS) were synthesized for this investigation, and their impact on the healing process of diabetic wounds was assessed. Using Design Expert, the electrospinning process for the fabrication of NS nanofibers (NS-NF) was adjusted for applied voltage (*X*_1_), the distance between the needle’s tip and the collector (*X*_2_), and the feed rate (*X*_3_) for attaining desired entrapment efficacy [EE] and average nanofiber diameter (ND). The optimal formulation can be prepared with 19.11 kV of voltage, 20 cm of distance, and a flow rate of 0.502 mL/h utilizing the desirability approach. All the selected parameters and responses have their impact on drug delivery from nanofibers. In addition, *M. sylvestris* extracts have been added into the optimal formulation [MS-NS-NF] and assessed for their surface morphology, tensile strength, water absorption potential, and in vitro drug release studies. The NS and MS delivery from MS-NS-NF has been extended for more than 60 h. *M. sylvestris*-loaded nanofibers demonstrated superior antibacterial activity compared to plain NS nanofibers. The scaffolds featured a broad aspect and a highly linked porous fibrous network structure. Histomorphometry study and the in vitro scratch assay demonstrate the formulation’s efficacy in treating diabetic wound healing. The cells treated with MS-NS-NF in vivo demonstrated that wound dressings successfully reduced both acute and chronic inflammations. To improve the healing of diabetic wounds, MS-NS-NF may be regarded as an appropriate candidate for wound dressing.

## 1. Introduction

Skin is the most significant vital organ of the human body and the first barrier against external pathogens (Nosrati et al., 2021). However, external mechanical forces, surgical operations, burns, chemical injuries, and ulcers from certain chronic diseases can cause varying degrees of damage to the skin (Fatehi & Abbasi, 2020). Wound healing is a complicated and dynamic tissue regeneration process composed of four stages: hemostasis, inflammation, proliferation, and remodeling (El Ayadi et al., 2020). Although the skin can undergo a certain degree of spontaneous repair, bacterial infection has always been the main reason hindering wound healing. An infected wound will not only disrupt the normal healing process but also cause the wound tissue to be deformed, causing great pain to the patient (Chen et al., 2019). Acute versus chronic wounds can be primarily categorized by their healing time. A wound that heals normally is acute, whereas a wound that is arrested in a phase of 70 healing is known as a chronic wound. A chronic wound’s inflammatory response is very different from a wound just starting to heal (Diegelmann, 2003). Inflammation in an acute wound removes necrotic tissue, debris, and bacterial contaminants from the wound bed and attracts and activates fibroblasts, the cells responsible for wound healing. By its very nature, inflammation is a self-limiting process under normal circumstances. On the other hand, chronic wounds are exacerbated by their inflammation, which serves no purpose other than to increase damage and inflammation. Neutrophils persist throughout the healing process in a chronic wound, but in an acute wound, they are primarily absent after the first 72 h (Lobmann et al., 2005). Neutrophils can stay around for a long time for several reasons, such as continued recruitment and activation due to tissue damage from pressure, bacterial overgrowth, leukocyte trapping, or ischemic-reperfusion injury.

Chronic wounds take a long time to heal because of inflammation, inadequate blood flow, and necrotic tissue. As a result, its damp, warm, and nutrient-rich atmosphere make it a perfect growing medium for microbes (Rath et al., 2016a). For certain wounds, microorganisms can colonize the wound bed and build a biofilm, which develops excellent resistance to antimicrobial agents and the immune system (Hill et al., 2010). In this regard, the production of antibacterial wound dressings is of the utmost importance. Antibacterial chitosan/polyvinyl alcohol/zinc oxide nanofibrous mats and alginate/silver/nicotinamide nanocomposites are developed to treat diabetic wounds (Montaser et al., 2016; Ahmed et al., 2018). During the past several decades, electrospinning is fast developing from a single-fluid blending process (Abd El Hady et al., 2021; Qi et al., 2021), to coaxial (Ning et al., 2021; Liu et al., 2022), triaxial (Liu et al., 2014), side-by-side (Wang et al., 2022), tri-layer side-by-side (Jiang et al., 2022a), and other complicated processes (Du et al., 2022), which greatly expand its capability of creating nanocomposites. However, nanocomposites often contain one drug for a designed drug-controlled release profile (Jaragh-Alhadad et al., 2022; Zhang et al., 2022). Multiple active ingredients can be simultaneously loaded into the nanofibers for a final functional performance (Zhao et al., 2022). Several recent reports summarized the loading of herbal compounds into nanofibers (Jiang et al., 2022a, 2022b).

Neomycin sulfate (NS) is one of the most commonly used topical antibiotics. It is the sulfate salt of neomycin B and C. It is an aminoglycoside antibiotic produced by the growth of *Streptomyces fradiae* (Nitanan et al., 2013; Geszke-Moritz and Moritz, 2016). It stops proteins from being made by binding to ribosomal RNA, which causes the bacterial genetic code to be read wrong. Except for *P. aeruginosa*, it kills most Gram-negative bacteria but does not affect anaerobes. Some Gram-positive bacteria, such as staphylococci, are killed by it, but streptococci are not. Neomycin is sold as 20% NS in petrolatum, and it is often mixed with other topical antimicrobials to make it more effective against Gram-positive bacteria. It can be used to treat superficial infections, prevent infections in minor wounds and postsurgery wounds, help treat burns, and deal with secondary infections in long-term skin conditions (Madan et al., 2014; Daneshmand et al., 2018; Paliwal et al., 2020). Even though it is often used to treat stasis dermatitis and chronic leg ulcers, it should be used with care because putting it on skin that is already damaged can cause sensitization, systemic absorption, and possibly systemic toxicity. Another harmful side effect of neomycin is allergic contact dermatitis, which affects 1% to 6% of the population with healthy skin and even more people with damaged skin. Contact dermatitis has been reported in as many as 30% of people with stasis dermatitis or leg ulcers. Neomycin can also cause delayed hypersensitivity, reactions caused by IgE, and anaphylactic reactions. The fact that neomycin could cause resistance is another drawback. Resistance can be caused by plasmids and has been seen in both Gram-positive cocci (like staphylococci) and Gram-negative cocci (like *Escherichia coli*, Klebsiella, and Proteus) (Madgulkar et al., 2011).

Herbal extracts have been studied extensively for their ability to speed the healing of wounds and reduce inflammation and pain (Gholamian-Dehkordi et al., 2017). Numerous electrospun matrices infused with herbal medications have been developed for skin tissue engineering (Merrell et al., 2009; Liakos et al., 2015; Mary & Dev, 2015). *Malva sylvestris* L. is grown as a medicinal plant in many different regions, including Europe, North Africa, and South and West Asia (especially Iran). The characteristics of *M. sylvestris* have been described in several scholarly publications. This plant’s healing effects come from the mucilage and flavonoids found in its leaves and petals. The plant displays properties such as antioxidant protection, inflammatory suppression, cancer prevention, wound healing, hepatoprotection, antinociception, and antimicrobial protection (Kurakula & Naveen, 2020a; 2021).

Researchers report accurate analysis results of *M. sylvestris extracts* (Gasparetto et al., 2012). Historically, *M. sylvestris* extract has been used topically to promote healing after wounds. Also, *M. sylvestris* cream is made, and its effects on rats with burns are studied (Afshar et al., 2015). The *M. sylvestris* cream was found to enhance the histological changes of tissue components during the healing process. Alloxan-induced diabetic rats were given *M. sylvestris* to see if it has any wound-healing properties (Pirbalouti et al., 2010).

However, a review of the literature revealed that no studies on optimizing process parameters for manufacturing NS nanofibers had been conducted. Furthermore, the integration of *M. sylvestris* extracts into this nanofiber was not studied. In this study, chitosan nanofibers containing NS and *M. sylvestris* extract were developed as a hydrophilic wound dressing to extend and improve the transport of herbal components to diabetic wounds.

## 2. Materials and methods

### 2.1. Materials

Yarrow Chemicals, Mumbai, India generously gifted NS. Dried *M. sylvestris* flowers were procured from a local herbal drug market (Maddur, Karnataka, India) and was identified. Polyvinyl alcohol (PVA) and chitosan ([2-amino-2-deoxy-(1-4)-β-d-glucopyranose], with medium molecular weight, 400,000 Da) were purchased from Sigma Aldrich, USA. All other chemicals and solvents used were of analytical grade.

### 2.2. Compatibility studies

#### 2.2.1. Fourier-transform infrared spectroscopic studies

Fourier transform infrared spectroscopy measures the absorption of infrared radiation by the sample material versus wavelength. The infrared absorption bands identify molecular components and structures. Infrared spectroscopy was performed using a Thermo Nicolet FTIR, and the spectrum was recorded between 4000 and 400 cm^−1^. By measuring any shift in the peaks of the drug in the spectrum of a physical mixture of drugs, IR-spectral experiments revealed the interaction between drugs and excipients (Begum et al., 2017; Aldawsari et al., 2022; Sreeharsha et al., 2022). The pellets were made on a KBr-press (Spectra Lab, India), and an infrared spectrophotometer was used to look at them.

### 2.3. Preparation of NS nanofibers

Briefly, 3.5 g of PVA (10% wt/wt in water), 1 g of chitosan (3% wt/wt in HCl 0.5 M), and 0.5% of NS were irradiated under ultrasonic for 30 min. The prepared solution was stirred for 7 h at 80 °C. The electrospinning of the final prepared solution was performed at various electrical voltages (15–20 kV) at room temperature under atmospheric pressure. The polymer fibers were injected using a 5-mL syringe needle having a 1.23 mm outer diameter, and 0.83 mm internal diameter, and the distance between the tip of the syringe and collector was optimized along with different flow rates.

#### 2.3.1. Experimental design

The preparation of NS nanofibers (NS-NF) is standardized by the statistical model RSM. The voltage applied (*X*_1_), distance (*X*_2_), and flow rate (*X*_3_) were chosen as individual parameters at three distinct levels encoded as – 1 (low), 0 (medium), and +1 (high). These parameters were scrutinized for their influence on entrapment efficacy [EE] and average nanofiber diameter (ND) using Box–Behnken Design (BBD) of Design Expert Version 12 (Stat Ease Inc., USA), originating 17 experiments runs (Naveen et al., 2020; Kurakula & Naveen, 2020b). Table 1 represents the total experiment plan, coded, and actual values of chosen parameters, and the responses’ restrains. Analysis of variance (ANOVA) was used to validate the developed polynomial equations. Also, various statistical tools were employed in all test runs to select the best-fit model. A quadratic design was employed in every test run to quantify the outcome response and regression analysis.

$$Y_{i(\mathrm{Quadratic})}=b_{0}+b_{1}X_{1}+b_{2}X_{2}+b_{3}X_{3}+b_{4}X_{1}X_{2}+b_{5}X_{1}X_{3}+b_{6}X_{2}X_{3}+b_{7}X_{1}^{2}+b_{8}X_{2}^{2}+b_{9}X_{3}^{2}$$

where, *Y_i_* – chosen response or dependent variable, *b*_0_ – computed response and *b_i_* – The estimated coefficient for main effects (*X*_1_, *X*_2_, *X*_3_); interaction terms of main effects (*X*_1_*X*_2,_ *X*_2_*X*_3_, *X*_1_*X*_3_), and polynomial terms of independent variables ($X_{1}^{2}$, $X_{2}^{2}$, $X_{3}^{2}$).

**Table 1.** Experimental plan for Box-Behnken design in terms of actual and coded values.

	Levels				
Factors/Independent Variables	–1	0	+1	Responses/dependent variables	Constraints
Voltage (kV) – *X*_1_	15	17.5	20	EE	Maximum
Distance (cm) – *X*_2_	10	15	20	ND	Minimum
Flow rate (mL/h) – (*X*_3_)	0.4	0.5	0.6		

### 2.4. Characterization

#### 2.4.1. Nanofiber diameter

Scanning electron microscopy (Hitachi S-4200) was used to characterize the morphology of the electrospun nanofiber scaffolds. The samples were coated with silver and examined at an accelerating voltage of 15 kV (Zhijiang et al., 2016; Cam et al., 2021). The fiber diameter distributions of the scaffolds were obtained by analyzing the SEM micrographs by image-analysis software (Adobe Photoshop 7.0). One hundred fibers were measured for each sample using four SEM images.

#### 2.4.2. Entrapment efficacy

The drug loading capacity is the ratio of the bound drug mass to the scaffold’s mass (Pandey et al., 2011). Whereas drug entrapment efficiency is the ratio of the mass of the drug released to the total drug added. The drug loading capacity of nanofibrous scaffolds was determined by measuring the concentration of the unbound drug present in the supernatant using UV–vis spectra at 292 nm. The NS concentration in the supernatant was evaluated using a standard calibration curve. Briefly, 20 mg of the scaffold was dissolved in 2 mL of phosphate buffer saline (pH 7.4), and then the sample was centrifuged at 8000 rpm for 5 min. After four times centrifugation, the supernatants were collected together, 3 mL of ninhydrin reagent was added, and the NS content was quantified using a Lambda 25 UV − vis spectrophotometer (Perkin-Elmer, USA) at 292 nm with a concentration/absorbance calibration curve at the same wavelength. The drug loading capacity was calculated using the equation

EE (%) =T−F/T × 100

where, *F* = the free amount of NS in the supernatant, *W* = the weight of the scaffold, and *T* = the total amount of NS.

### 2.5. Extraction of plant material

The dried flowers of *M. sylvestris* (700 g) were ground into a powder and extracted using the maceration method with ethanol and distilled water (80:20) at room temperature. The maceration method was done three times for a total of 72 h and 6 L of solvent. The extracts were mixed, strained through a paper filter, and dried at 40 °C under a vacuum. The reproducibility of the extract has been confirmed on a trial-and-error basis. In addition, several phytochemical methods were adopted to identify several functional groups, and the results were in accordance with the previously reported studies (Benso et al., 2015; Fahimi et al., 2015; Fathi et al., 2021; Nozohour and Jalilzadeh, 2021).

### 2.6. Preparation of M. sylvestris extarct laoded NS-NF [MS-NS-NF]

For the preparation of extract-loaded nanofibers, 10% wt/wt of the extract was added to the mixtures, which were then stirred for one hour before electrospinning. Then, the prepared solutions were electrospun with the optimized parameters described earlier.

### 2.7. Tensile test

The tensile strength of nanofibrous scaffolds was measured and evaluated using an Instron 4411 tensile test equipment, and the data were analyzed using Bluehill 2 software (Elancourt, France). Six samples 10 × 50 mm in size were collected from each group, and the thickness of each sample was measured using a digital micrometer (Mitutoyo MTI Corp., USA) to determine the tensile strength of the samples. All samples were put in with care at both ends of the system. Each sample was put through a tensile test at a speed of 5 mm/min and with 1 cm between the grips (Lee et al., 2003a).

### 2.8. Water uptake capacity

O-NS-NF and MS-NS-NF were subjected to water uptake capacity (WUC) studies. Briefly, 50 mg of electrospun nanofibrous scaffolds were put in distilled water at 37 °C. After 0.5, 1, 2, 3, 8, and 24 h, samples were obtained from the water. First, the surface water of the samples was removed using filter paper, and then the samples were weighed. The WUC (g/g) was found by weighing the nanofibrous scaffolds before (*W*_b_) placing them in water for different amounts of time and right after (*W*_a_) taking them out of the water, as shown in the following equation

$$\text{WUC}\;\left(g/g\right)=\left(\left(W_{a}-W_{b}\right)/W_{b}\right)$$

### 2.9. In vitro drug release study

In vitro drug release was done to evaluate the release of NS and the herbal extract from fabricated O-NS-NF and MS-NS-NF (Eltayeb et al., 2016; Huo et al., 2021). A standard curve for NS was made to study its release characteristics. A standard solution of pure NS in a mixture of 91% water and 9% ethanol (vol/vol) was made, and then it was changed. A UV–vis spectrophotometer was used to measure the absorbance of the solution at 292 nm. The phosphate buffer solution was used for the study of release. At 37 °C, a certain amount of O-NS-NF was put into 50 mL of PBS. At regular intervals, 1 mL of PBS solution was taken out and replaced with the same amount of fresh PBS to keep the volume the same. Then, a derivatization treatment was done to the extracted solution, and absorbance was measured with a UV–vis spectrophotometer. A standard curve was used to figure out how much NS was released and then plotted against time for up to 32 h. All of the measurements were done three times. The amount of NS released was figured out using a standard curve, which was then plotted against time for up to 32 h. Three times each measurement was taken. The same methodology has been applied to study the release of NS and MS from MS-NS-NF till 84 h.

### 2.10. Porosity measurements

Archimedes’ principle and fluid displacement measurement techniques were used to figure out how porous the scaffold was (Lee et al., 2003b). In short, each disc-shaped scaffold (10 mm in diameter and 5 mm thick) was put in a graduated cylinder with a known amount of ethanol (*V*_1_). After putting the scaffold in water (*V*_2_), the total volume was measured. After an hour, the scaffolds were taken out along with the solvent that had been trapped in the pores. The amount of ethanol left in the cylinder was written as *V*_3_. The equation *V*_T_ = *V*_2_ – *V*_3_ (Murugan & Ramakrishna, 2006; Chong et al., 2007) was used to figure out the scaffolds’ total volume (*V*_T_). The porosity was then found by using the following equation to figure out the amount of space in the material compared to the total volume of the scaffold (*n* = 5):

$$\text{Porosity}\;\%=V_{1}-V_{3}/V_{T}\;\times \;100\%$$

### 2.11. Assessment of antibacterial activity

The antibacterial activity of nanofibers against Gram-positive *Staphylococcus aureus* (S. aureus, ATCC 6538) and Gram-negative *Escherichia coli* (E. coli, ATCC 8739) was evaluated using the previously reported colony counting technique (Yu et al., 2012). Counts of bacterial colonies (CFU) were taken.

Antibacterial activity (%)=(B−T/T) × 100

where, *T* = cfu*/mL of the test sample, cfu = concentration of colony of bacteria, *B* = blank sample and cfu* = colony-forming unit. The agar dilution method determined the extracts’ minimum inhibitory concentrations (MIC).

### 2.12. MTT assay

The cell culture study was done according to the procedure described by Zahedi et al. (2013). In this procedure, mesenchymal stem cells were isolated from the matrix of a human umbilical cord (hUCM). When the concentration of mesenchymal stem cells reached about 80% (1 × 10^6^), the samples were combined with the culture medium. Using 3-4,5-dimethylthiazol-2-yl-2,5-diphenyltetrazolium bromide, the wound dressing samples were tested to see if they were compatible with cells (MTT).

### 2.13. In vitro scratch assay

The scratch experiment was carried out using Balb/3T3 fibroblast cell lines. To replicate diabetes conditions, cells were cultured in DMEM with 10% PBS and 50 mM glucose. The cells were then spread out in a single layer on the tissue culture plates at 70%–80% confluence. A scratch was made on the plate with a 1 mL pipette tip. Different test samples were made in the new medium, put on plates, and kept in an incubator for 24 h. The cells were then washed, and a 4% formaldehyde solution was used to treat them (Bobadilla et al., 2019).

### 2.14. Preparation of diabetic rats and evaluation of wound healing

In the diabetic study, male Wister rats weighing 150 and 200 g were used. The rats were housed at standard room temperature (23 °C) and on a 12 h light/dark cycle, with full access to rodent food and water. All experiments were conducted in conformity with the ethical guidelines for the care and use of laboratory animals [SACCP-IAEC/2020-01/18]. Rats developed diabetes after receiving a single intraperitoneal injection of sterile streptozotocin (Sigma, St. Louis, MO, USA) in sodium citrate (0.1 mol/L, pH 4.5). Animals with blood glucose levels over 300 mg/dL have diabetes. Animals were made unconscious by intraperitoneal injections of xylazine (13 mg/kg) and ketamine (66.7 mg/kg) (Almasian et al., 2020). The dorsal hair of rats was shaved, and a 1.5-cm-diameter, full-thickness incision was made using a biopsy punch. The rats were randomly divided into three groups of five. Groups A and B were established for the wounds of animals treated with both formulations. The control rats in Group C received a regular gauze bandage. Photographs were taken at different time intervals to evaluate diabetic rats’ wound healing. Using an analytical computer system, the nonhealing part of the wound was identified. Using the following formula, the rate of recovery was determined:

Healing rate (100%) = primitive area – nonhealing area/primitive area × 100

### 2.15. Histomorphometry analysis

Semiquantitative evaluation of epithelialization was performed on a 5-point scale on day 14: 0 (no new epithelialization), 1 (25%), 2 (50%), 3 (75%), and 4. (100%). In addition, sections were assessed semiqualitatively in terms of angiogenesis, which was scored on a 5-point scale based on the number of new blood vessels inside the scar tissue: Zero (none), one (few), two (moderate), three (many), or four (many) are not present (considerably). A comparative study undertaken by a single, independent observer who was uninformed of the treatment groups verified the results for these measures. In addition, Image-Pro Plus® V.6 was utilized to compute and evaluate neovascularization and collagen density for histomorphometric analysis (Media Cybernetics, Inc., Silver Spring, USA).

### 2.16. Statistical analysis

All results were compared using Kruskal Wallis analysis. Results with *p* < .05 were considered statistically significant. Statistical analyses were performed using the SPSS software version 20.0 (SPSS, Inc., Chicago, USA).

## 3. Results and discussion

**Figure 1.**
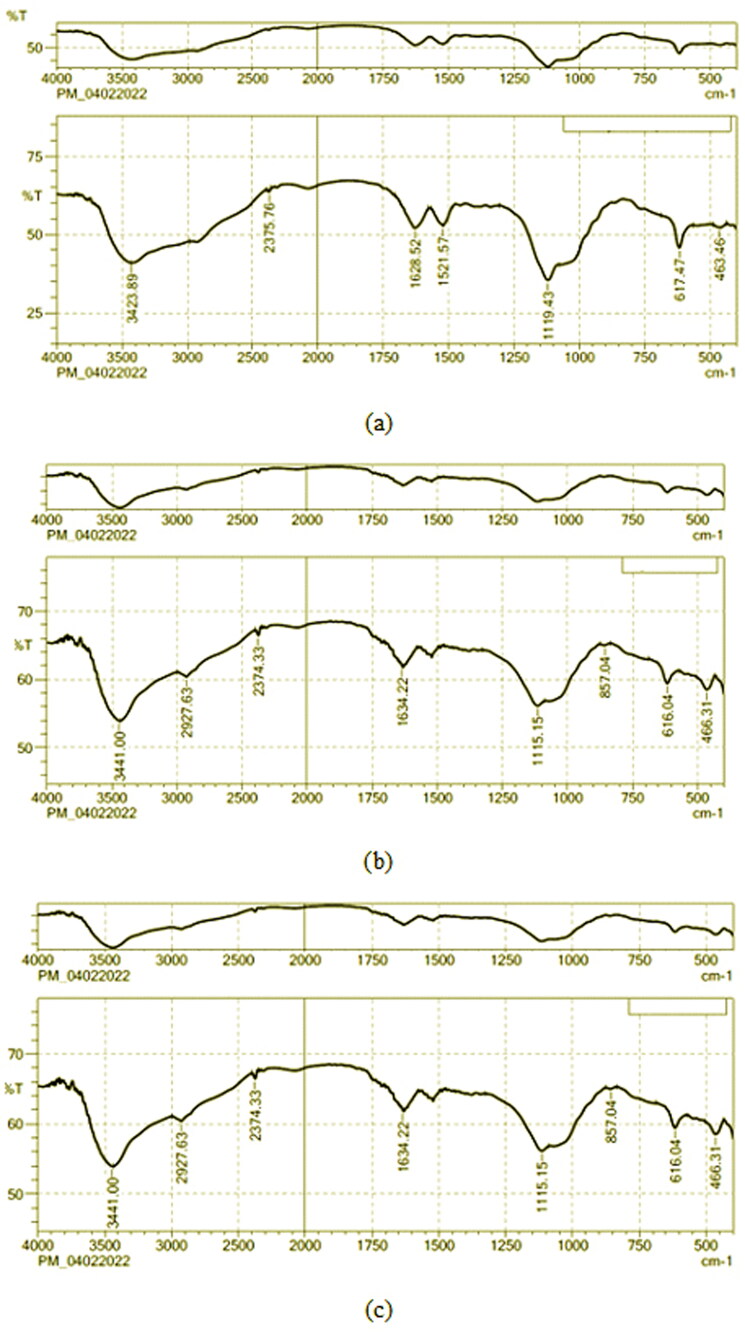
FTIR spectrum of (a) NS, (b) physical mixture of optimized formulation of NS-NF, (c) physical mixture of optimized formulation of NS and M. sylvestris [MS-NS-NF].

Figure 1(a,b) shows the characteristic band of NS and the physical mixture of the optimized formulation, respectively. The stretching vibrations of NH groups were linked to the bands at 3423.89 cm^−1^. Also, the bands at 3782.24 cm^−1^ and 2348.74 cm^−1^ were attributed to the stretching vibrations of OH stretching and C-H stretching (Aromatic), respectively. At 1521.57 and 3898.75 cm^−1^, C = C and C-H stretching were seen. At 1628.52 and 1119.43 cm^−1^, C-O and C = O stretching were seen. In the end, all these bands were found in the physical mixture of NS and the excipients.

When the extract was added, the bands between 1000 and 1200 cm^−1^ became stronger. This showed that the extract might have interacted with the ether groups of polymers through hydrogen bonds. Also, the band at 3423 cm^−1^ was moved up to 3441 cm^−1^ with a higher wavenumber. The bands at 1119 and 1521 cm^−1^ were moving toward lower wavenumbers. Also, compared to the NS curve, the strength of the bands at 2927 and 1628 cm^−1^ (which have to do with the bending vibration of OH groups) was lower (Hosny et al., 2022). The bending vibrations of CeH groups in phenyl rings caused the peak to show up at 857 cm^−1^. The peak near 616 cm^−1^ is due to CH vibrations that are out of plane in ethylene systems. All of these interpretations of the spectrum showed that the pure NS and the chosen ingredients worked well together (Figure 1(c)).

### 3.1. Preparation of NS-NF

PVA and chitosan were used in many medical situations because it is biocompatible, don’t stick to proteins very well, and aren’t toxic. So, an electrospinning instrument was used to make nanofibers from NS (selected antibiotic), PVA, a natural polymer (chitosan), and a plant extract. BBD was used to find out how certain variables and how they affect each other effect the minimum ND and maximum EE. There were 17 planned experiments, and their results are shown in Table 2. From 194 to 401 nm, the ND of experimental formulations was found. EE, which will be used to determine how much drug was entrapped, was found to be between 69.54 and 95.21%. fx model and ANOVA were used to look at all of the experiment results for specific responses.

**Table 2.** The results of predicted experimental runs are observed.

		Factor 1	Factor 2	Factor 3	Response 1	Response 2
Std	Run	*A*: Voltage (kV)	*B*: Distance (cm)	*C*: Flow rate (mL/h)	EE (%)	ND (nm)
4	15	20	20	0.6	77.94	194
1	10	15	10	0.6	76.74	228
3	7	15	20	0.6	73.61	254
6	6	20	15	0.4	77.58	275
2	1	20	10	0.6	82.69	287
5	17	15	15	0.4	75.05	305
7	8	15	15	0.8	69.54	307
10	3	17.5	20	0.4	88.78	324
9	9	17.5	10	0.4	91.28	378
15	4	17.5	15	0.6	94.68	381
16	14	17.5	15	0.6	95.21	382
12	11	17.5	20	0.8	79.29	384
14	16	17.5	15	0.6	93.78	384
13	13	17.5	15	0.6	94.25	385
17	12	17.5	15	0.6	93.41	387
8	2	20	15	0.8	72.84	394
11	5	17.5	10	0.8	84.25	401

On the basis of the sequential sum of squares (Type-I) and the fit summary, the quadratic model was chosen for all replies. For model selection, *F*-value, *p* value, and *R*^2^ values were evaluated. In addition, the quadratic model has the most significant polynomial order with a *p* value (degree of statistical significance) of 0.0001 (Table 3).

**Table 3.** Model statistical summary.

Response	Models	*R* ^2^	Adju. *R*^2^	Pred. *R*^2^	Adequate precision	Sequential *p* value	Remarks
EE	Linear	0.1185	–0.0850	–0.4132	–	.6370	
	2 FI	0.1203	–0.4075	–1.7238	29.2609	.9992	
	Quadratic	**0.9926**	**0.9831**	**0.9047**	–	**<.0001**	Suggested
	Cubic	0.9984	0.9936		–	.0803	Aliased
ND	Linear	0.1147	–0.0895	–0.6691	–	.6497	
	2 FI	0.2199	–0.2482	–2.2435	–	.7235	
	Quadratic	**0.9910**	**0.9794**	**0.8607**	28.200	**<.0001**	Suggested
	Cubic	0.9997	0.9987		–	.0025	

The discrepancy between the predicted *R*^2^ of 0.9047 and the adjusted *R*^2^ of 0.9926 is less than 0.2 for EE. Adeq precision measures the signal-to-noise ratio. A ratio larger than four is preferred. The ratio of 29.2609 is indicative of a sufficient signal. This approach facilitates design space navigation. Similar results were found for ND [0.8604, 0.9794, and 28.200] (Naveen et al., 2020). The normal plot of residuals further demonstrated the correctness of all chosen models. As the visual inspection graph is satisfactory, the prescribed statistical application will not be implemented. For each of the selected answers, the distribution of the studentized residuals was closer to the straight line, indicating that the proposed model may be accepted statistically (Jeirani et al., 2012; Liu & Ho, 2017). Supplementary Figure S1 identifies the experimental run against the residuals as a method for identifying the factors that influence the answers hiding in plain sight. A dispersed trend was identified within the specified boundary, indicating a background time-coupled variable slink. The coefficient of variation (CV) value confirms that the reproducibility of the tests not only assures the accuracy of the findings but also the clarity of the approach, as demonstrated by the repeatability of the experiments. As needed CV value was relatively low (1.39 percent for EE and 2.84 percent for ND) compared to the mandated (CV 10 %), consistency and accuracy of the design were ensured. A further parameter, Lack of Fit, evaluates the model’s incapacity to account for all the data (Chen et al., 2015). As seen by the ANOVA results, the Lack of Fit is non-significant (*p* > .05), confirming the suitability of the chosen design. ANOVA was used to examine the quantitative impacts of certain variables on responses (Rizg et al., 2022). Multiple regression was used to the collected data to generate polynomial equations. The Model *F*-values of 104.50 and 85.60 indicate that all chosen models are statistically significant.

EE, *A*, *B*, *C*, *AB*, *A*2, *B*2, and *C*2 are crucial model terms in this scenario. The experimental design suggested that EE might be altered by (i) the antagonist effect of factor *B*, *C*, and all polynomial terms of A, B, and C; and (ii) the synergistic effect of factor *A*, with *A*^2^ having the strongest effects compared to all other terms. The experimental design suggested that ND might be altered by (i) the antagonistic impact of factor *B*, *AB*, and the polynomial terms of *A* and *B*; and (ii) the synergistic effect of *C*, *AC*, and the polynomial terms of *C*, with *A*^2^ effects being the most significant (Table 4).

**Table 4.** Analysis of variance (ANOVA) results.

	Intercept	*A*	*B*	*C*	*AB*	*AC*	*BC*	*A*²	*B*²	*C*²
EE	94.266	2.01375	–1.9175	–3.34625	–0.405	0.1925	–0.615	–14.3343	–2.18675	–6.17925
*p* values		.0017	.0023	<.0001	.5081	.7500	.3248	<.0001	.0062	<.0001
ND	383.8	7	–17.25	25.5	–29.75	29.25	9.25	–97.275	–45.775	33.725
*p* values		.0745	.0013	.0001	.0004	.0005	.0913	<.0001	<.0001	.0002

Equations generated for coded factors,

$$\text{EE}\;=+94.27\;+2.01\;A-1.92\;B-3.35\;C-0.4050\;AB\;+0.1925\;AC-0.6150\;BC-14.33\;A^{2}-2.19\;B^{2}-6.18\;C^{2}$$

$$\text{ND}\;=+383.80\;+7.00\;A-17.25\;B\;+25.50\;C\;-29.75\;AB\;+29.25\;AC\;+9.25\;BC\;-97.28\;A^{2}-45.78\;B^{2}+33.73\;C^{2}$$

**Figure 2.**
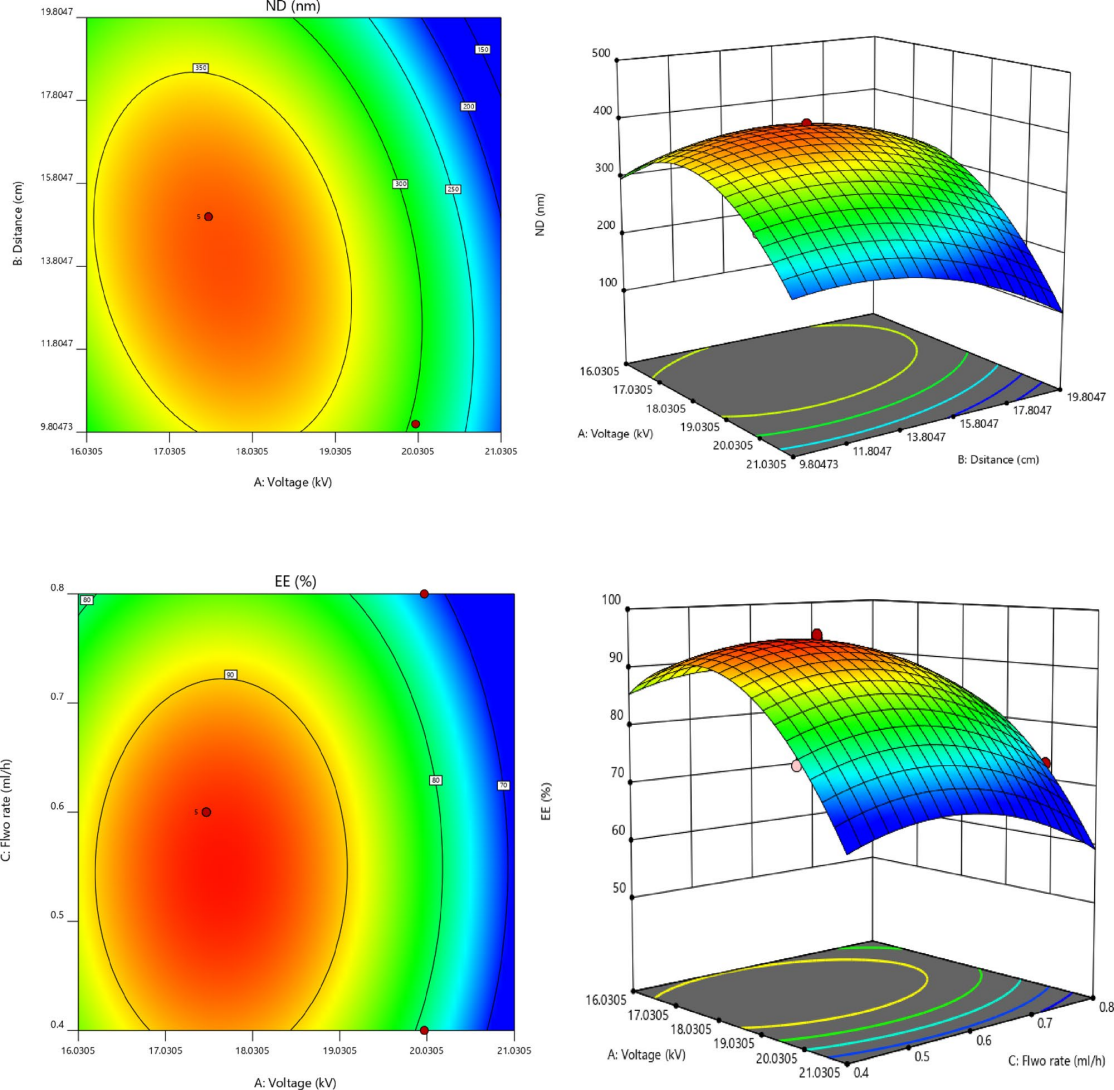
Contour and response surface plots for EE and ND.

All of the aforementioned equations may be used to predict the response for any concentration of the specified components. In addition, factor coefficients aid in comparing the relative influence of factors on answers. Contour plots and 3D RSG (response surface graphs) are necessary to describe the interaction and main effect, and (Figure 2) illustrates the observed responses using these graphs.

**Figure 3.**
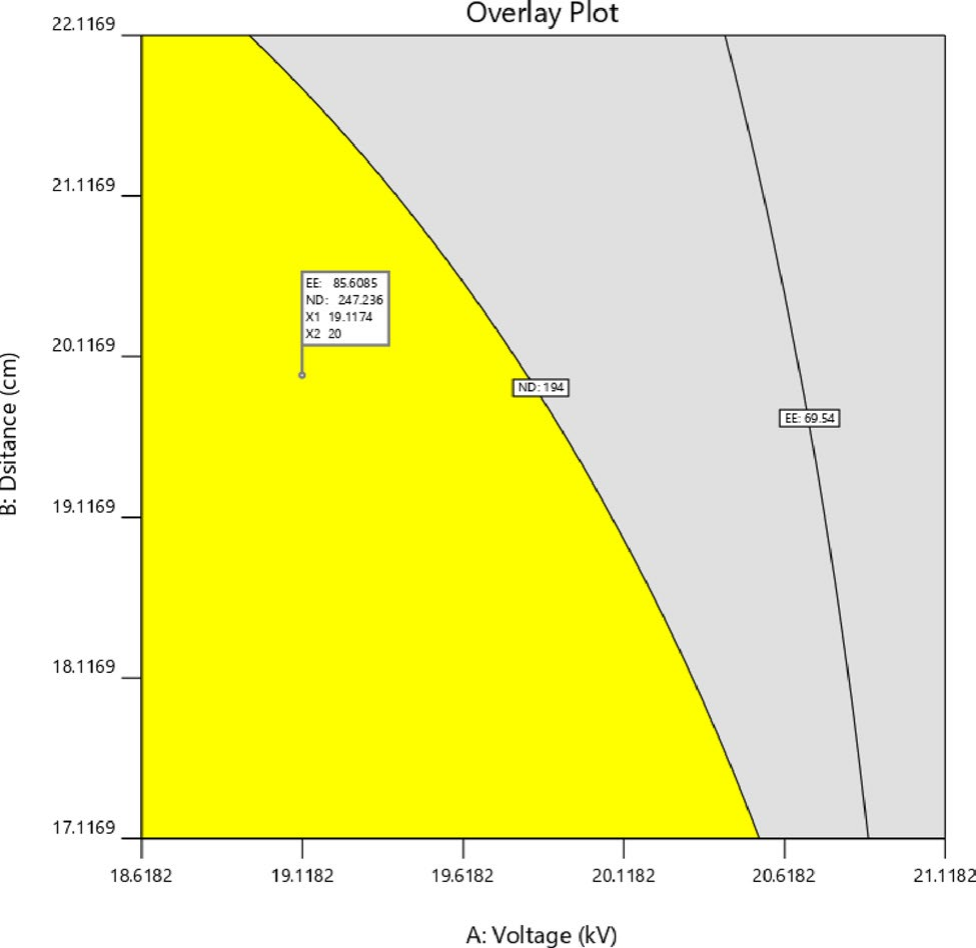
Overlay plot of optimized formula.

Applying the desirability function [*D*] enables the optimization of various models derived from the experimental investigation. To produce the overlay graph, each response was adjusted to different limits, particle size, and zeta potential-maximum and PDI-minimum. The design space included all of the chosen variables. The combined desirability plot for all answers revealed a maximum *D* value of 0.859, which was attained at optimal concentrations of independent variables, and the contour plot for the critical responses (Figure 3) was superimposed with these responses. Utilizing this method to desirability, a formulation made using 19.11 kV of voltage, 20 cm of distance, and a flow rate of 0.502 mL/h may meet the requirements of the optimum formulation. Therefore, employing these optimum concentrations may result in an EE of 85.6066% and an ND of 247.208 nm. Using these anticipated optimal concentrations, an improved formulation of O-NS-NF was formulated and assessed. To verify the experimental design, theoretical values were compared to experimental data. Less than 3% relative inaccuracy was observed, confirming the accuracy of the design (Naveen et al., 2017).

**Figure 4.**
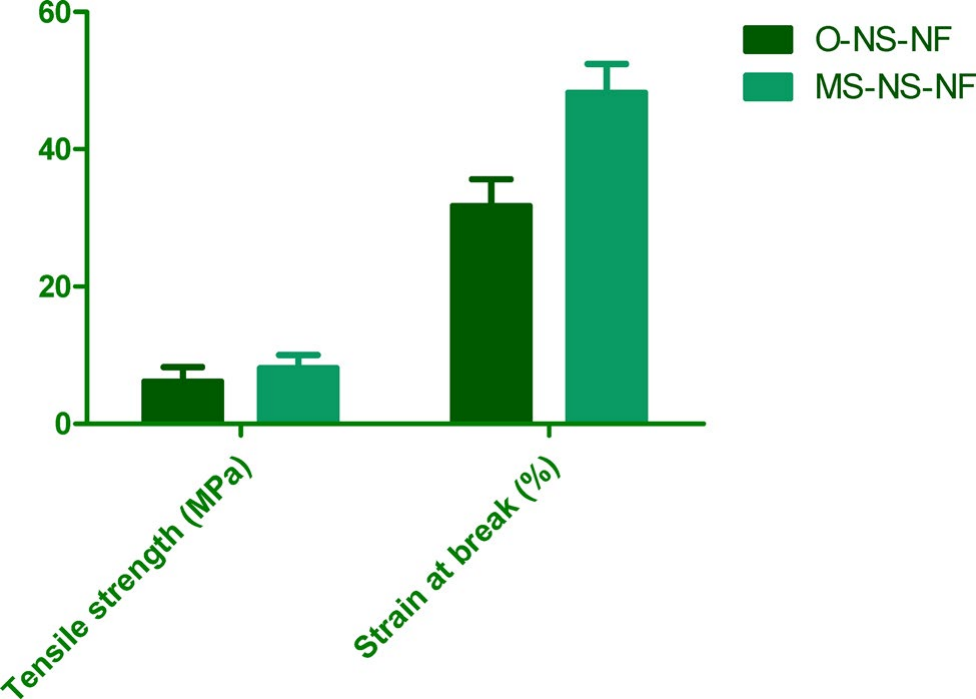
Comparison of tensile strength and strain at break for prepared formulations.

Using a tensile tester, the mechanical characteristics of O-NS-NF and MS-NS-NF samples were determined. Figure 4 shows the tensile strength and strain at failure for each sample. According to the experimental findings, MS-NS-NF samples exhibit higher tensile strength and stronger durability than O-NS-NF samples. When medication and MS were added to NS-NF, the tensile strength increased relative to NS alone. Consequently, the addition of MS to the composite makes it stronger. Similar outcomes and tendencies were seen with the strain at break.

#### 3.2.1. Water u ptake

**Figure 5.**
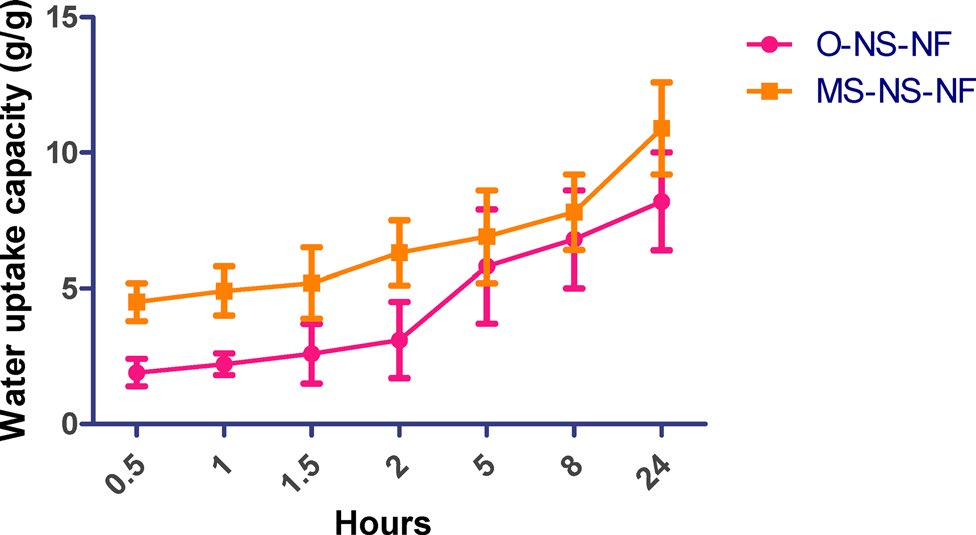
Water uptake study of O-NS-NF and MS-NS-NF.

In general, rat wound healing occurs by contraction, but a sequential cellular signaling process can be observed in human wound healing. Hence this study connects the importance of the water uptake study in the human wound healing process. To facilitate nutrition transport and cell signal transduction, nanofibrous scaffolds with a favorable water-holding capacity absorb wound exudate and maintain a moist environment. Consequently, cell growth and proliferation are enhanced, and wound healing is expedited. Therefore, the water-holding capacity of scaffolds was examined as an essential parameter (López-Vélez et al., 2003). Within 24 h, nanofibrous scaffolds expand quickly as immersion time increases. The water absorption content of samples MS-MS-MF and O-NS-NF rose throughout time and peaked at 10.9 and 8.2 g/g, respectively, 24 h after sampling (Figure 5). Due to the high PVA content in composite fiber, the water absorption values of O-NS-NF samples were relatively low, rendering them incapable of retaining water. In addition, it is shown that MS-NS-NF nanofibrous scaffolds have a favorable water retention capacity and can absorb at least eight times their initial weight in 24 h. It was evident that the inclusion of MS improved water uptake.

#### 3.2.2. Drug release

**Figure 6.**
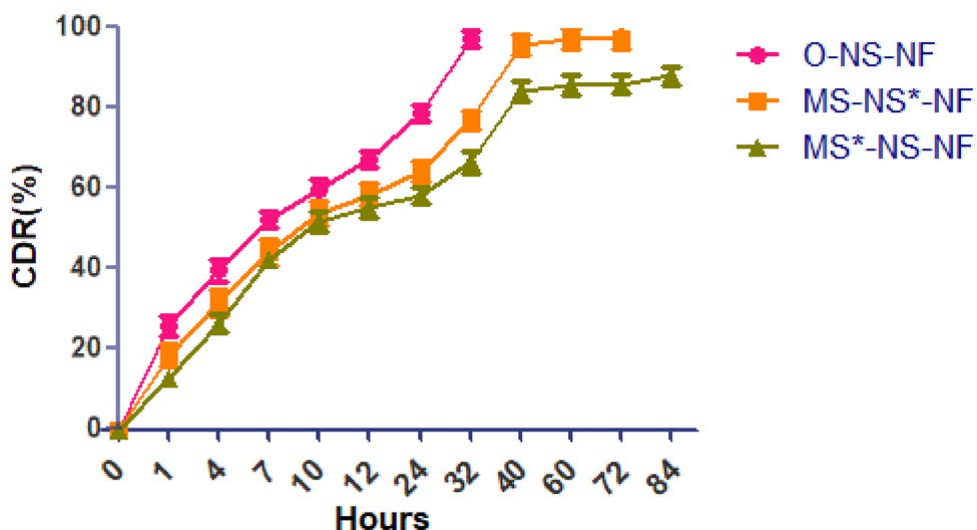
In vitro drug release profile of O-NS-NF and MS-NS-NF [MS-NS*-NF represents the release of NS and MS*-NS-NF- shows the release of Malva sylvestris extract].

In vitro, drug release tests were performed for both formulations. Before calculating the in vitro drug release, linear standard calibration curves for NS (data not shown) were constructed, and then the collected information was utilized to calculate drug release. UV spectroscopy was used to determine the amount of released herbal compounds at the maximum wavelength of 323 nm. It is reported that phenolic compounds identified with a maximum in the 300–550 nm range presumably arise from the B-ring, a band I and Band II, with a maximum in the 240 to 285 nm range which is believed to arise from the A-ring (López-Vélez et al., 2003). The UV–visible spectrum of *M. sylvestris* in phosphate-buffered saline was recorded (the figure is not shown). The spectra were seen to have a strong and clear peak at 323 nm. The UV–visible spectra of solutions containing various extract amounts were analyzed. All spectra revealed that the peak at 323 nm was crisp, prominent, and unique. Consequently, it was chosen for detecting phenolic chemicals. After that, 84 h of in vitro drug release studies were conducted. All samples releasing profiles were determined in phosphate-buffered saline (PBS) at pH 7.4 (Figure 6). Except for the first two hours, NS from O-NS-NF demonstrated sustained drug release from the beginning to the conclusion of the drug release tests. It may be attributable to the polymer composite’s greater chitosan content. After this phase, the release rate was lowered during the next 16 h and achieved equilibrium after 40 h. At equilibrium, the residual quantity of chemicals released from nanofibers was close to zero. This shows that herbal components are released more slowly from nanofibers. It was discovered that increasing nanofiber diameter significantly decreased the quantity of extract released by nanofibers. However, the quantity of extract released remains high for the first 24 h. According to the statistic, the percentage of data released in the first stage reduced from 64.28 to 58.25%. This was owing to the reduced swelling ratio of nanofibers generated with MS compared to nanofibers with an optimal formulation. Also, the rate of extract release reduced after 36 h, and equilibrium was reached after 60 h. At the same time, NS and MS release form the best formulation (MS-NS-NF) was more than for 60 h and then followed by very slow phase. MS release was almost near to 72–84 h. NS release was extended beyond 60 h. The first excerpt is to be released. The release behavior was consistent with the water swelling profiles, demonstrating that the swelling water ratio mostly governed herbal ingredient release through diffusion. The larger swelling ratio of the nanofibers is attributable to a greater quantity of water penetrating the polymer matrix, which results in enhanced solubility of herbal components.

#### 3.2.3. Surface morphology and porosity

**Figure 7.**
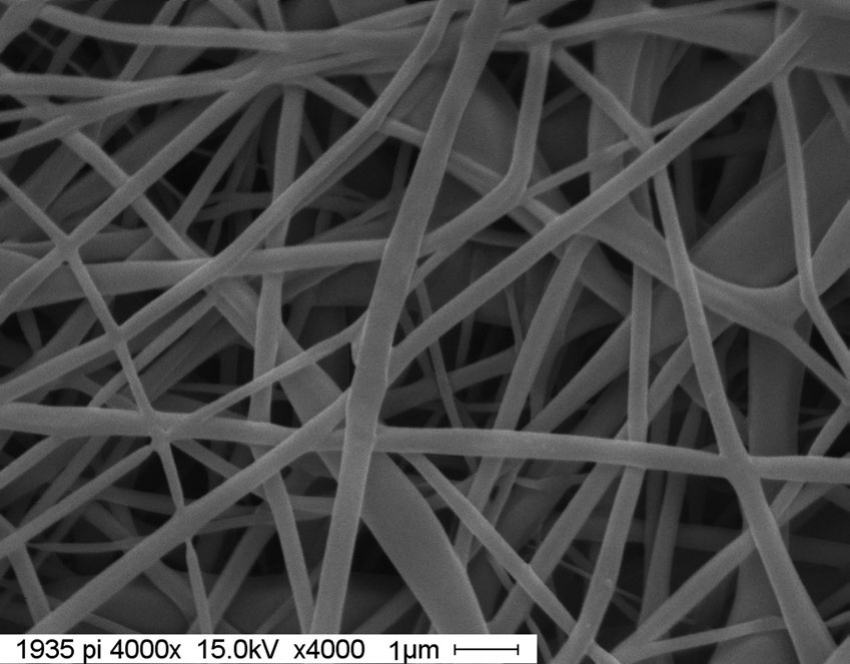
Surface morphology of MS-NS-NF.

Figure 7 depicts the surface morphology of electrospun MS-NS-NF nanofiber scaffolds. It is possible to detect the porous structure, and the nanofibers have a cylindrical, homogeneous, bead-free, and random orientation. The porosity of the MS-NS-NF was found to be 69.25%. By examining SEM micrographs using image-analysis tools, the diameter of each fiber was determined (Adobe Photoshop 7.0). 100 fibers were counted from four SEM pictures for each sample. In O-NS-NF and MS-NS-NF nanofiber scaffolds, the average nanofiber diameter is 248.20 nm and 165.35 nm, respectively. MS-NS-NF nanofiber scaffolds may have a smaller diameter and narrower diameter dispersion owing to the low-molecular weight of the components.

#### 3.2.4. Antibacterial activity

The antibacterial investigation was conducted using colony-forming units, an effective technique for determining the antimicrobial ability of any molecule. In contrast to the MS-N S-NF nanofibers, the O-NS-NF nanofibers exhibited weaker antibacterial activity against *S. aureus* and *E. coli*. The antibacterial activity of a nanofiber-containing herbal extract against *S. aureus* was 69.85%. In addition, the antibacterial activity against *E. coli* levels was 70.69%, respectively. Malvone A is responsible for the antibacterial properties of the *M. sylvestris* flower extract (Pirbalouti & Koohpyeh, 2010). Malvone A, (2-methyl-3-methoxy-5, 6-dihydroxy-1,4-naphthoquinone), also has several exceptional features, such as anti-inflammatory, excretion of free radicals, and antioxidant activity, making it an excellent option for the treatment of wounds. In general, phenolic chemicals contributed considerably to the antibacterial activity of the plant extract (Pirbalouti & Koohpyeh, 2010). The total phenolic content of *M. sylvestris* extract was 8.225 mg GAE/g, as evaluated by Folin Ciocalteu reagent. Total phenolic content significantly depends on the extraction solvent, water content, and duration.

#### 3.2.5. MTT assay

**Figure 8.**
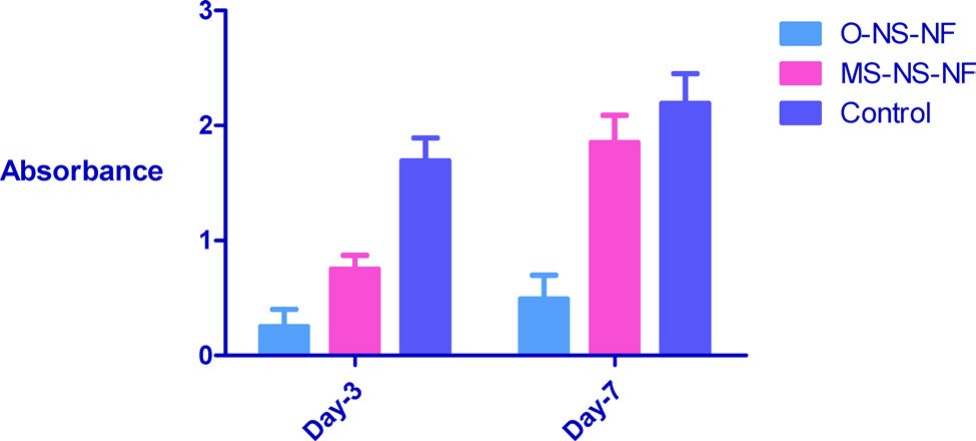
The viability of wound dressings after 3 and 7 days.

MTT test was used to determine the nontoxicity of wound dressing. Figure 8 depicts the vitality of mesenchymal stem cells on wound dressings. The absorbance readings indicated an increase of 88.8% after seven days, showing that the produced wound dressing is nontoxic. After 7 days, the presence of mesenchymal stem cells on the wound dressings can be further analyzed using SEM.

#### 3.2.6. Scratch assay

**Figure 9.**
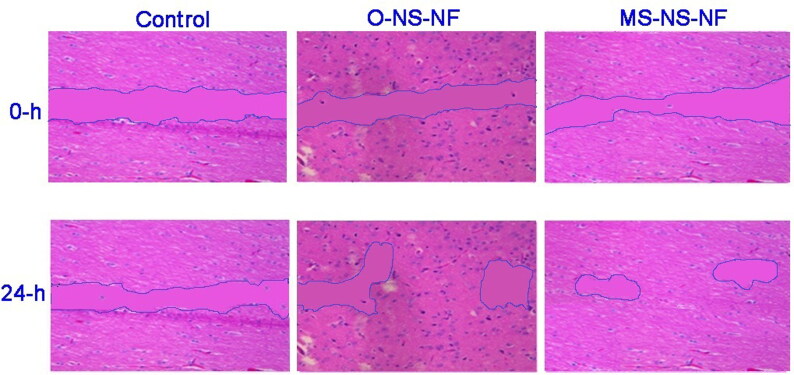
Migration of cells in Balb/3T3 fibroblasts cultured in the presence of the control, O-NS-NF, and MS-NS-NF.

The efficiency of O-NS-NF and MS-NS-NF scaffolds to promote in vitro wound closure was employed to examine the impacts of Balb/3T3 fibroblast cells migratory capabilities. They monitored the pace of wound closure for 24 h. Cells treated with MS-NS-NF scaffold moved more rapidly than those treated with O-NS-NF or a control (Figure 9). This proves the effectiveness of loading MS in addition to NS.

##### 3.2.7. Wound healing activities

**Figure 10.**
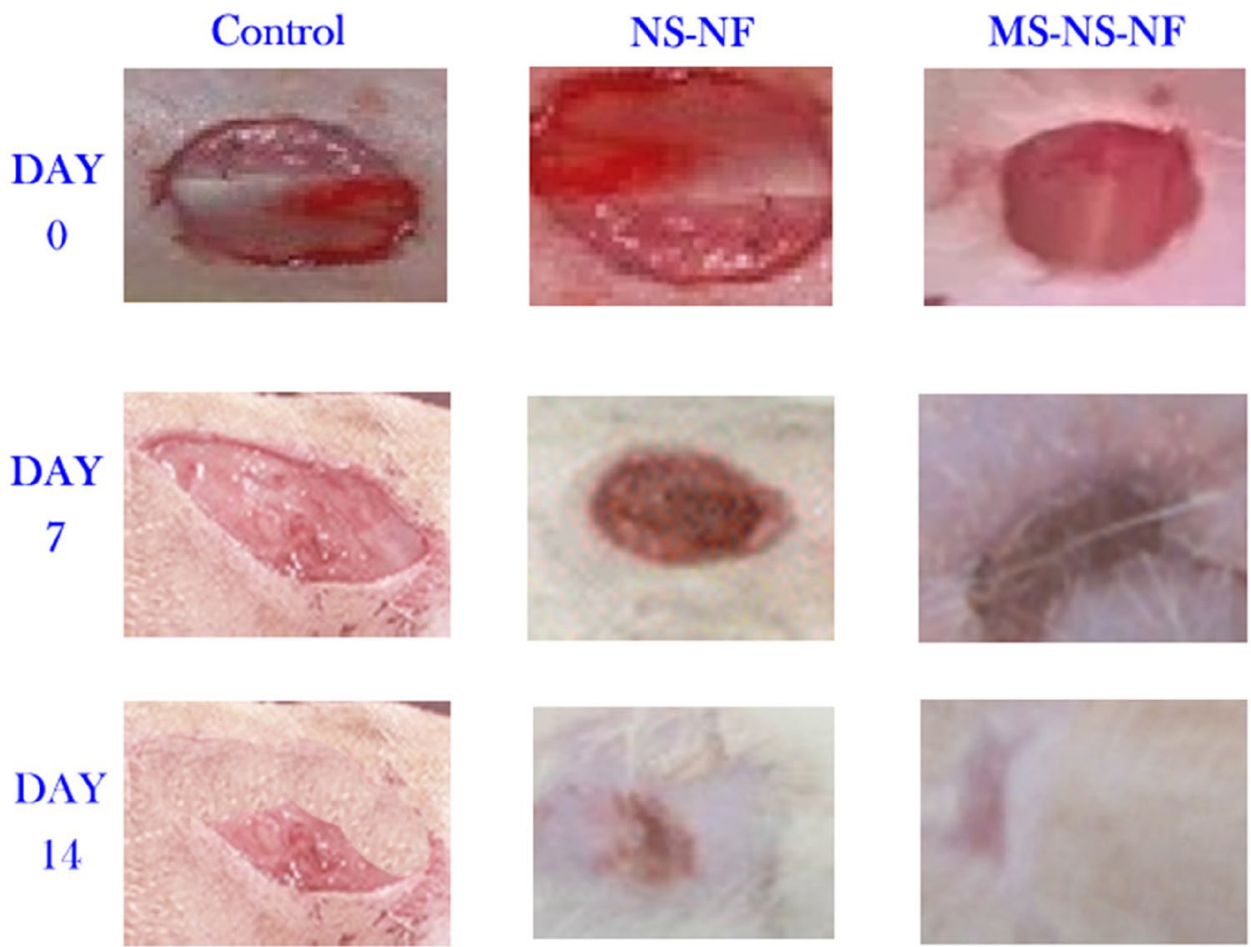
Representative wounds on an animal in each group on days zero, 7, and 14 days after treatment.

Damaged blood and lymphatic vessels release a fluid, which hemostasis counteracts when the skin is injured. Platelets begin to consolidate in the wound bed and form a clot to occlude the defect region due to growth factor secretion (Cordeiro & Jacinto, 2013). Following this, platelet chemokines activate the inflammatory cells, initiating the inflammatory phase (Darby et al., 2014). During this phase, growth factors and cytokines are secreted, macrophages and other immune cells are activated and travel toward the wound to eliminate cell debris and combat invading germs. Ultimately, keratinocyte proliferation starts re-epithelialization (Vukelic et al., 2011). During the period of proliferation, fibroblasts develop into myofibroblasts, and tissue granulation begins. Myofibroblasts break down the extracellular matrix (ECM) and create a new ECM (Zahedi et al., 2013). Figure 10 depicts a typical wound on an animal from each group on days 0, 7, and 14 after treatment. On day 7, diabetic rats treated with a basic gauze bandage, NS-NF, and MS-NS-NF had a wound healing percentage of 32.1%, 54.85, and 67.28%, respectively. As observed, wounds covered with MS-NS-NF wound dressing healed faster than those covered with gauze. This may be attributed to the electrospun wound dressing’s increased fluid absorption value and bacteria barrier feature compared to the gauze bandage. At the time of the gauze bandage change, the absence of moisture on the surface of gauze-treated wounds caused some harm to the regenerated tissues, resulting in a low percentage of wound healing. It was evident from the picture that the lesion treated with an extract containing nanofibers healed more quickly than the wounds in the other groups, suggesting that the use of herbal extract enhanced wound healing via modifying inflammatory responses in a cutaneous dendritic cell line. During the first few days, most of the cells are made of inflammatory cells (Guarrera, 2005). Better granulation tissue formation and tissue repair was observed in MS-NS-NF than the other groups. Also, collagen deposition and neovascularization were higher in wounds treated with NS and MS nanofibers. The anti-inflammatory properties of *M. sylvestris* extract have been shown in the past. The pharmacological and biological action of *M. sylvestris* is associated with the presence of naphthoquinones, anthocyanins, flavonoids, and mucilaginous polysaccharides. The total flavonoid and anthocyanin concentrations of *M. sylvestris* extract were 20,477 mg CE/g and 0.78 mg Cg, respectively. However, at all observations, wound closure for all samples coated with an extract containing nanofibers had progressed more quickly than the others.

In addition, the size of wounds treated with an extract containing nanofibers was reduced. By day 14, the percentage of wounds treated with NS-NF and MS-NS-NF was found to be 89.64% and 96.08%, respectively, compared to 56.84% of wound healing in the control group. Wound contraction occurs throughout the healing phase owing to the proliferation of fibroblasts induced by contractile myofibroblasts. In accordance with the findings of the release and anti-bacterial tests, the proportion of wounds that healed in MS-NS-NF samples was found to be quite similar. Sustaining medication release after a successful first burst release is extremely desirable for the treatment of both the original and most recent wound infection (Rath et al., 2016b). On day 7, some inflammatory responses were reported for gauze-treated lesions. Blood vessels and granulation tissues were seen in the wounds treated with MS-NS-NF. Also evident was the development of fibroblast cells.

##### 3.2.8. Histomorphometric analysis

The findings of the histomorphometric investigation performed 7 and 14 days after skin damage are shown in Table 5. On day 14, angiogenesis, epitheliogenesis, and inflammatory cells indicated that the MS-NS-NF sample was superior to the other treatment [O-NS-NF].

**Table 5.** Histomorphometric analysis of three experimental groups.

Formulation		Angiogenesis (Score)	Epitheliogenesis	Inflammatory cells/3HPF
Control	7 Days	0	0	181
	14 Days	1	2	98
O-NS-NF	7 Days	2	0	109
	14 Days	2	4	48
MS-NS-NF	7 Days	3	1	95
	14 Days	3	4	31

## 4. Conclusion

Electrospun nanofibers are considered as ideal candidates for wound-healing application due to their superior properties such as efficiency as a bacterial barrier, mimicking extracellular matrix structure, and appropriate water vapor transmission rate. The electrospinning process was optimized based on the desirability approach in getting EE of 85.6066% and an ND of 247.208 nm. Using these anticipated optimal concentrations, an optimized formulation of O-NS-NF was formulated and further incorporated with *M. sylvestris* extract [MS-NS-NF]. The formulations were evaluated for tensile strength, MTT assay, release behavior, and antibacterial and wound healing properties. The results showed that adding MS extract in the polymer matrix enhanced nanofibers’ mechanical and water uptake. In vitro drug release studies confirmed the extended drug release of both constituents till 84 h of study. MTT analysis indicated the nontoxic nature of produced wound dressings. Also, the extract containing nanofibers showed 69.85% and 70.69% antibacterial activity against *S. aureus* and *E. coli* levels. In vitro scratch tests confirm that the cells treated with scaffold moved more rapidly than those treated with medication or with a placebo. The wound dressing containing herbal extract exhibited a wound healing rate of 96.08% by day 14. Thus, the *M. sylvestris* scaffold is bioactive and may be more suitable for cell proliferation (further studies may require to confirm the same), suggesting that this scaffold can be used for wound dressing or tissue engineering scaffolds.

This work was supported by the Deanship of Scientific Research, Vice Presidency for Graduate Studies and Scientific Research, King Faisal University, Saudi Arabia [Project No. GRANT748]. The authors acknowledge the funding support from Vidya Siri College of Pharmacy, India (No. VSCOP5/8/22). Part of this research was supported by the statutory activity of the Sri Adichunchanagiri College of Pharmacy, Adichunchanagiri University.

## Supplementary Material

Supplemental Material
