# Supplementary figures and images for: Optimization of process parameters for fabrication of electrospun nanofibers containing neomycin sulfate and Malva sylvestris extract for a better diabetic wound healing
Source: Drug Deliv. 2022 Nov 21;29(1):3370–83. doi: 10.1080/10717544.2022.2144963 (PMC9848420; doi:10.1080/10717544.2022.2144963)

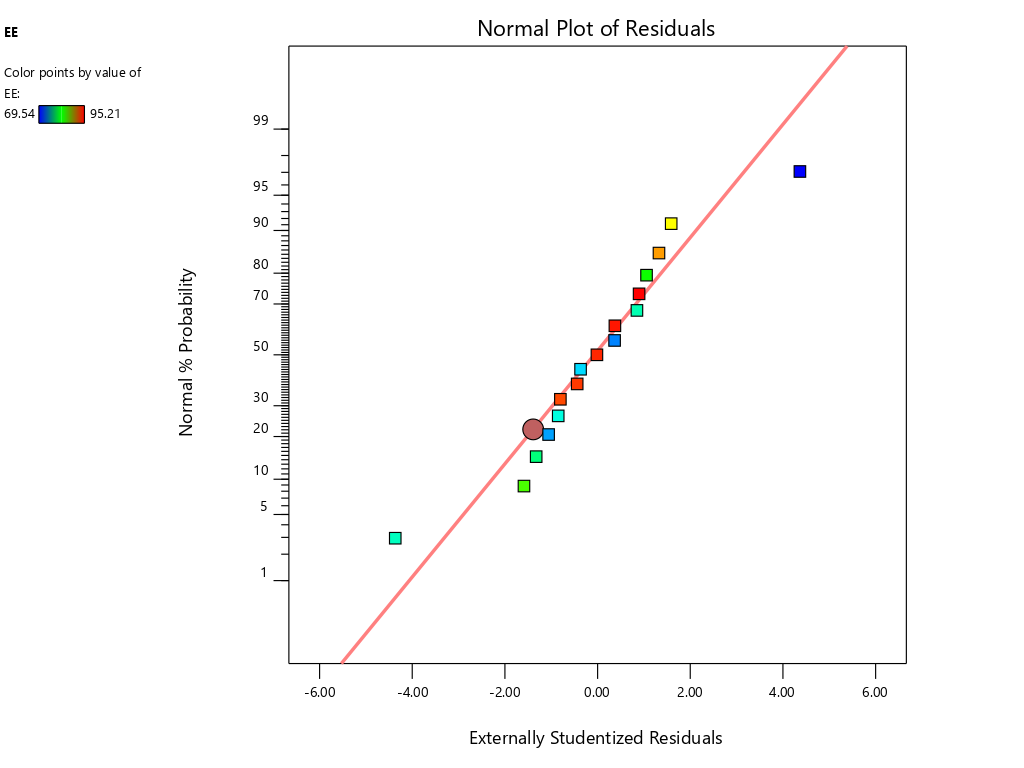

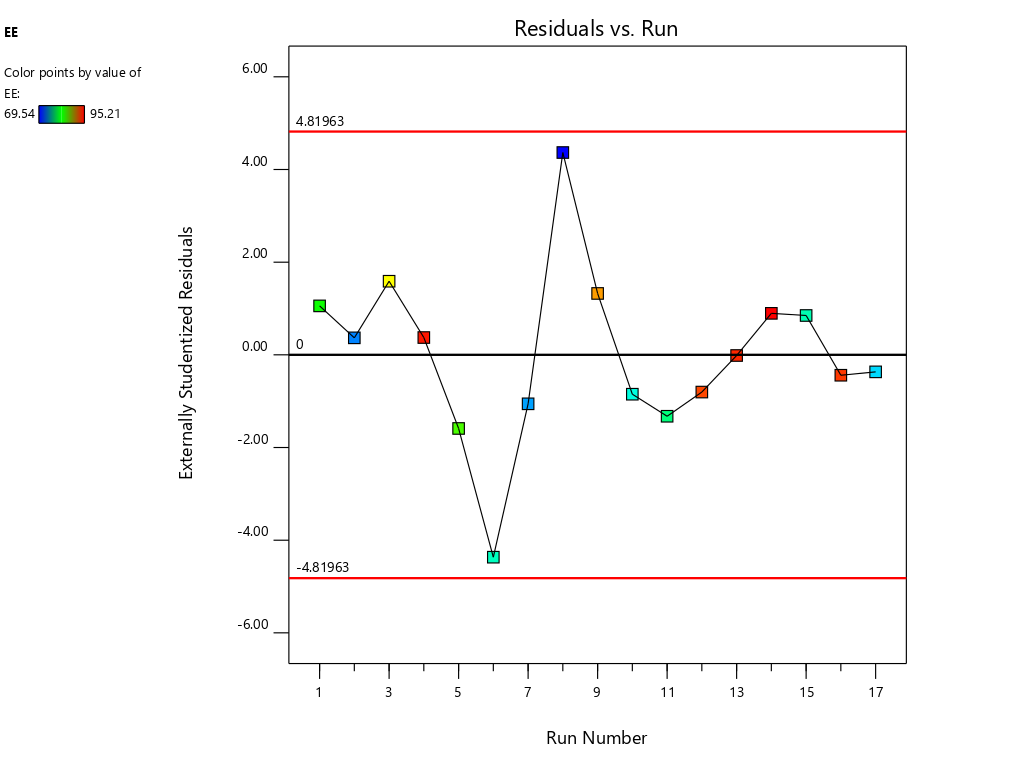


(a)


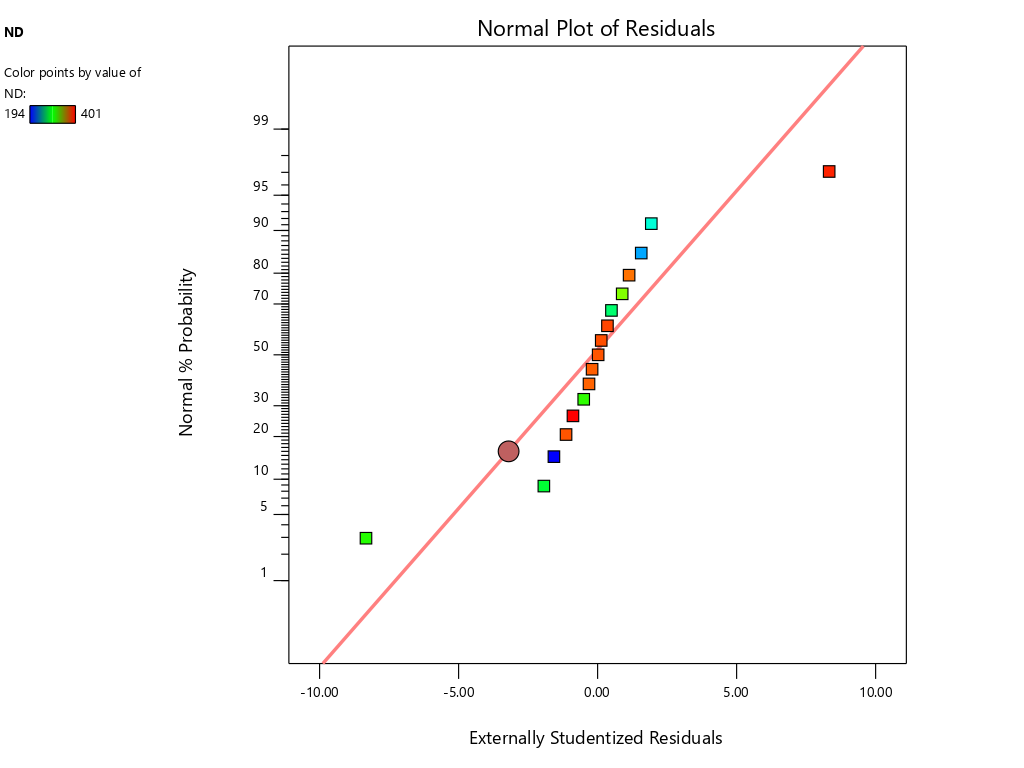


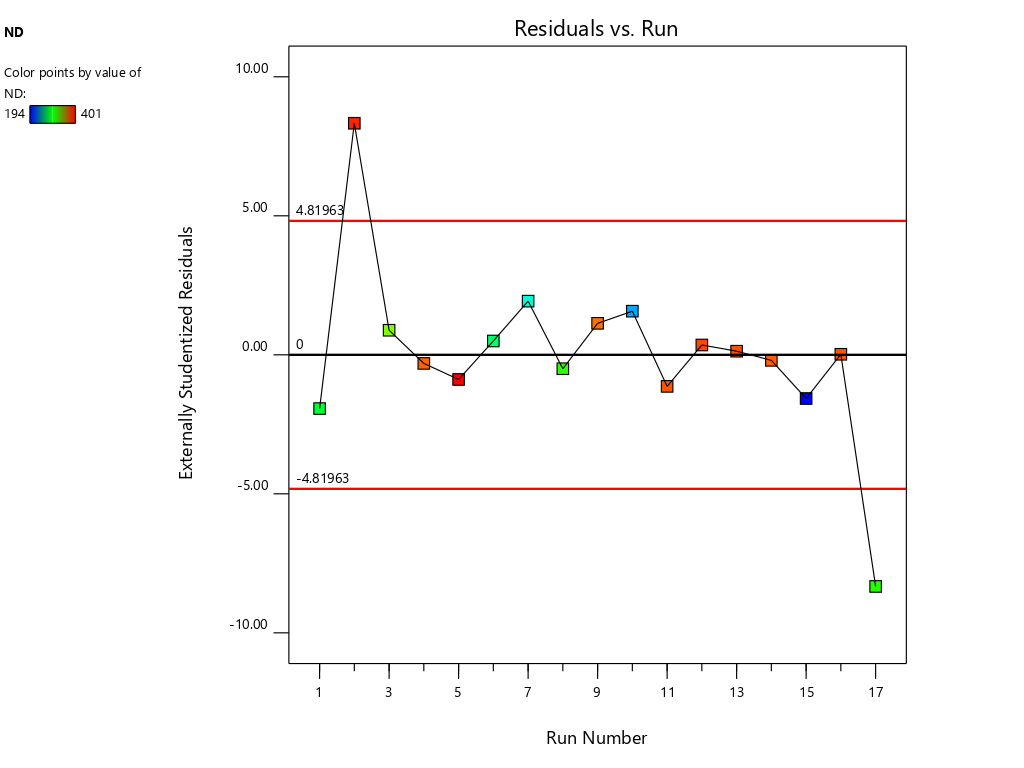


(b)

**Supplementary Figure 1.** The normal plot of residuals and residuals vs Run for EE and ND.

Supplement: Supplemental Material [file IDRD_A_2144963_SM4444.docx]
